# Supplementary material for: Pan-Cancer Analysis Reveals PPRC1 as a Novel Prognostic Biomarker in Ovarian Cancer and Hepatocellular Carcinoma
Source: Medicina (Kaunas). 2023 Apr 17;59(4):784. doi: 10.3390/medicina59040784 (PMC10146118; doi:10.3390/medicina59040784)
Supplement: Supplementary file 1 [file medicina-59-00784-s001.zip › medicina-2152176-supplementary.pdf]

Supplementary picture description

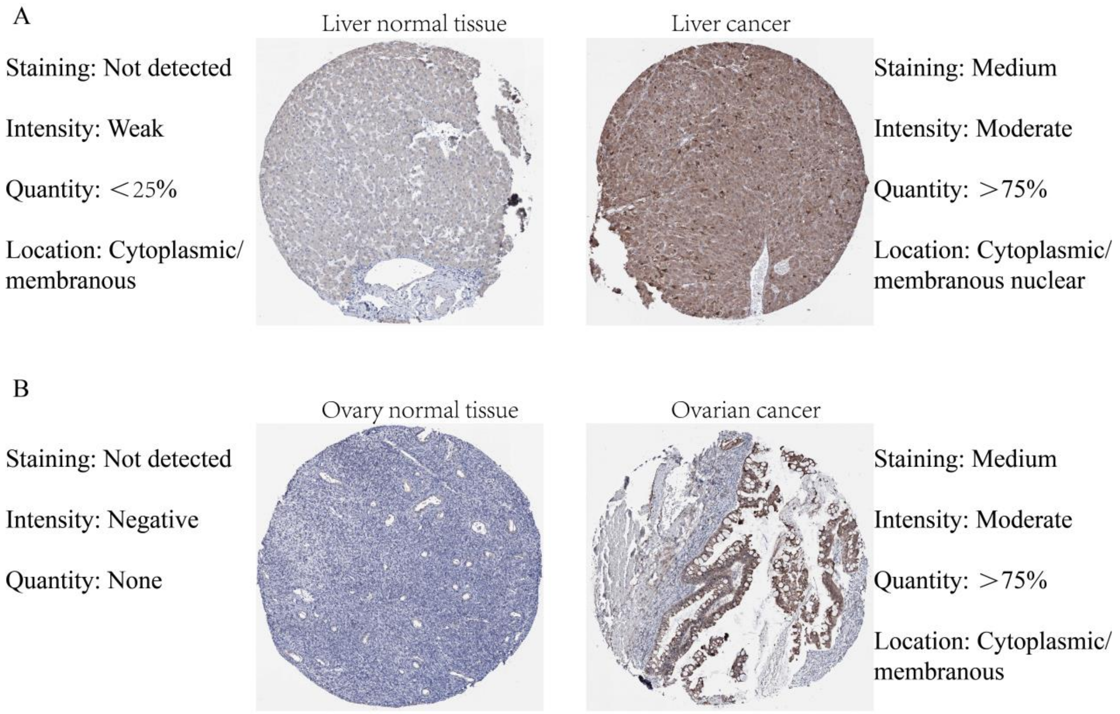

Figure S1.The differences in PPRC1 expression between normal tissues and ovarian cancer, liver cancer from the HPA database. The protein expression of PPRC1 in ovarian cancer were up-regulated compared with normal tissues (A) The protein expression of PPRC1 in liver cancer were up-regulated compared with normal tissues (B),IHC×100.

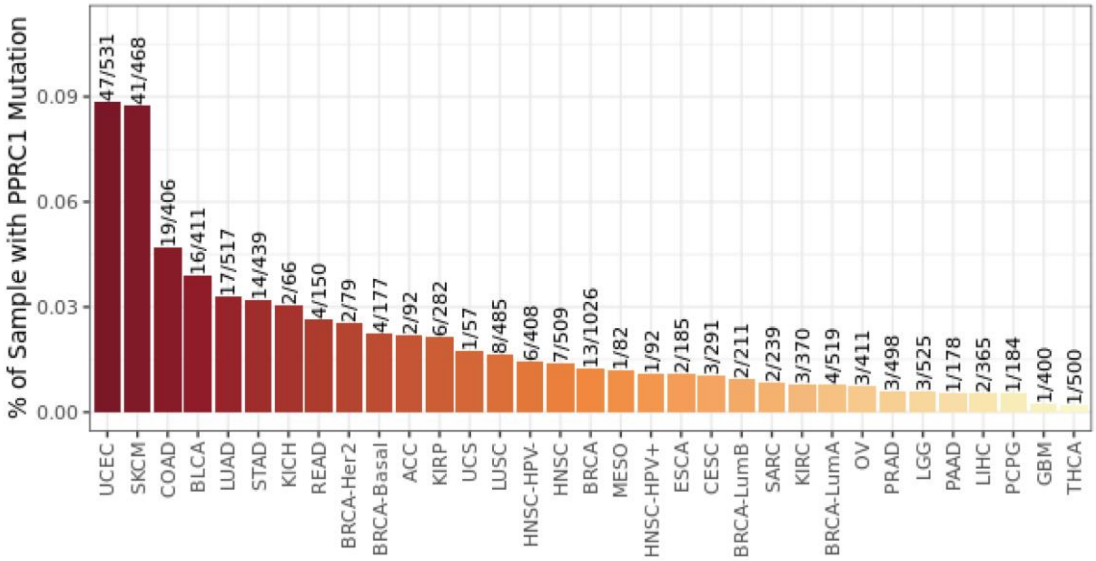

Figure S2.Correlation between expression of PPRC1 and mutation status in TIMER database.
